# RecRWR: A Recursive Random Walk Method for Improved Identification of Diseases

**DOI:** 10.1155/2015/747156

**Published:** 2015-03-22

**Authors:** Joel Perdiz Arrais, José Luís Oliveira

**Affiliations:** ^1^Department of Informatics Engineering (DEI), Centre for Informatics and Systems of the University of Coimbra (CISUC), University of Coimbra, 3030-290 Coimbra, Portugal; ^2^Department of Electronics, Telecommunications and Informatics (DETI), Institute of Electronics and Telematics Engineering of Aveiro (IEETA), University of Aveiro, 3810-193 Aveiro, Portugal

## Abstract

High-throughput methods such as next-generation sequencing or DNA microarrays lack precision, as they return hundreds of genes for a single disease profile. Several computational methods applied to physical interaction of protein networks have been successfully used in identification of the best disease candidates for each expression profile. An open problem for these methods is the ability to combine and take advantage of the wealth of biomedical data publicly available. We propose an enhanced method to improve selection of the best disease targets for a multilayer biomedical network that integrates PPI data annotated with stable knowledge from OMIM diseases and GO biological processes. We present a comprehensive validation that demonstrates the advantage of the proposed approach, Recursive Random Walk with Restarts (RecRWR). The obtained results outline the superiority of the proposed approach, RecRWR, in identifying disease candidates, especially with high levels of biological noise and benefiting from all data available.

## 1. Introduction

A major research domain in molecular biology is the study of the causal association between genomic variations and clinical phenotypes [1–3]. Classical methods use a manual approach where one or a limited number of genomic targets are individually tested. However, due to the resources needed to systematically perform this procedure and due to the difficulty in controlling all experimental variables, improved strategies were required. The possibility to use computational methods to identify the best disease candidates to be further validated was a major breakthrough [4–8]. A common constraint of most methods is the need for training data, which is scarce and difficult to validate.

A recent research trend consists of exploiting the topological properties of protein-protein interaction (PPI) networks combined with other biological data to envisage the underlying mechanisms of genetic diseases. Barabási et al. [9] and Joy et al. [10] discuss the role of proteins with high betweenness as mediators of relevant metabolic processes. Ma and Zeng [11] explore the use of the closeness centrality to quickly identify the top central metabolites in large scale networks. Approaches proposed by Erten et al. [12] and Arrais and Oliveira [13] explore the potentialities of the nodes with high degree for the prioritization of disease-associated genes.

While the previous methods focus on evaluation of the weights given to each node, a complementary strategy consists of evaluating the proximity of two given nodes in the network. Some common methods to conduct this task are the shortest path, log-likelihood, propagation matrix, and the RWR (Random Walk with Restarts). Previous studies confirm that the RWR outperforms other methods [14–18]. One common limitation of these studies is that they assume the graph is single concept, meaning that every node is equally treated. However, as we demonstrate in this study, those methods are poor when the graph integrates nodes from distinct data types.

In this paper, we propose a novel method to improve selection of the best disease targets for multiconcept graphs. Towards this aim we build a multilayer biomedical graph that stores PPI data, annotated with stable knowledge from OMIM diseases and Biological Process from Gene Ontology. The inherent improvements of the proposed method are (a) the use of multilayer networks formed with PPI data and by the terms' associations; (b) the combination of this data to establish new associations among nodes; and (c) the use of degree-based methods to evaluate node weights.

Finally, we present comprehensive validation that demonstrates the superiority of the proposed approach, Recursive Random Walk with Restarts (RecRWR).

## 2. Methods

The method proposed herein uses a graph representation of biomedical knowledge centred on proteins enriched with biomedical terms. The first step consisted of selecting and curating the required data and using it to construct the graph. For performance issues this network is represented as a matrix of adjacencies. Based on this ground-based biomedical graph we apply a modified version of the Hubs and Authorities (HITS) [13] algorithm adapted to this particular subject, in order to obtain a normalized and more accurate association among relations. Although here we are interested in tuning to protein-disease association, it is important to stress that it can be extended to the study of general association of many-to-many biomedical terms. Finally, we formulate how the proposed method, RecRWR, can be applied to this subject.

### 2.1. Multiconcept Graph Modelling

A graph-based representation is used to store the relations among the biomedical terms. Since we are integrating three distinct data sources, three interconnected subgraphs are obtained.

- PPI data are retrieved from STRING database [19], where the average confidence level is considered. A filter is applied to select only human.
- Disease data are extracted from OMIM morbid map [20] data where the genotype-phenotype associations are preserved. The morbid map is also used to extract the mapping relation for known protein diseases.
- Biological Process from Gene Ontology (GO) [21] Directed Acyclic Graph structure is extracted and replicated. The GO-GO mapping is also retrieved and stored.

For each of the previous data sources a curated set of terms $\vec{a}$ is extracted:

(1) $$\begin{array}{c} \vec{a}=\left(a_{1},a_{2},\ldots ,a_{P}\right), \end{array}$$

with *a*_*k*_ representing the content of the *a*th term from the interval (*k* ∈ [1, *P*] ⊂ ℕ). Each term *a*_*k*_ is a tuple of three elements that can be represented as

(2) $$\begin{array}{c} a_{k}:\left\{t_{a},t_{b},w\right\}, \end{array}$$

where the element *t*_*a*_ has an association with the element *t*_*b*_, with a confidence score *w* where (*a*, *b* ∈ [1, *R*] ⊂ ℕ) and (*w* ∈ [0,1000] ⊂ ℕ).

The set of vectors $\vec{a}$ are modelled as a nonoriented weighted graph *G* = (*V*, *E*, *W*).

- Each vertex *v*_*x*_ ∈ *V* is obtained by identifying the unique entry *t*_*a*_-*t*_*b*_ or *t*_*b*_-*t*_*a*_ of all the association tuples contained in vector $\vec{a}$. The vertices are labelled by their unique identifier.
- Each edge *e*_*x*_ ∈ *E* connects vertexes (*v*_*m*_, *v*_*n*_) representing an association between the terms represented by the vertices *v*_*m*_ and *v*_*n*_ contained in vector $\vec{a}$.
- The weight *w*_*v*_*m*_,*v*_*n*__ of each edge *e*_*x*_ corresponds to the score between two nodes.

The graph *G* = (*V*, *E*, *W*) is then mapped to an adjacency matrix representation that consists of a |*V*| × |*V*| = *n* × *n* matrix *A*:

(3) $$\begin{array}{c} A=\left(\begin{array}{ccc} a_{11} & \cdots & a_{1n}\\ \vdots & \ddots & \vdots \\ a_{n1} & \cdots & a_{nn} \end{array}\right),\;n=\left|V\right|,\;\;a_{i,j}\;\in \left[0,1000\right]\subset N. \end{array}$$

Because the graph is undirected the adjacency matrix is symmetric and therefore *a*_*ij*_ = *a*_*ji*_.

The compiled graph resulted in 60.000 nodes with an average degree of 5. The memory space required to represent the graph is Θ(|*E*|), which is realistically equivalent to a memory space of 6.0 MB, excluding hash tables required for node mapping. The adjacency matrix requires a memory space of Θ(|*V*|^2^), 7.2 GB.

### 2.2. RecRWR: Recursive Random Walk with Restarts

Next we formulate the RecRWR algorithm including a detailed pseudocode description of the algorithm (Algorithm 1). The three main components are

- Random Walk with Restarts;
- recursive cross subgraph mapping;
- node replacement.

### 2.3. Random Walk with Restarts

The final probability vector of Random Walker is defined as

(4) $$\begin{array}{c} {\vec{p}}^{t+1}=\left(1-r\right)W{\vec{p}}^{t}+r{\vec{p}}^{0}, \end{array}$$

where *W* is the column-normalized adjacency matrix *A* and ${\vec{p}}^{t}$ is a vector in which the *i*th element holds the probability of being at node *i* at time step *t*. The vector *p* ^0^ holds the probability of the initial states and is constructed such that equal probabilities are assigned to the list of seed nodes where the sum of the probabilities is equal to 1. This is obtained by a given list *L* of seed nodes, where *L* ⊂ *V*.

### 2.4. Recursive Cross Subgraph Mapping

We extend the previous formulation to a symmetric matrix composed of *k* ^2^/2 of submatrixes, where *k* corresponds to the number of data sources. The submatrix that corresponds to the mapping between the subgraphs *i* and *j* is obtained by

(5) $$\begin{array}{c} W_{ij}=\mathrm{diag}\left(\vec{m_{i}}\right)*W*\mathrm{diag}\left(\vec{m_{j}}\right), \end{array}$$

where $\vec{m_{i}}$ and $\vec{m_{j}}$ are binary vectors with n elements that represent the mask of the source and target subgraphs where $i,j\in \left[1,k\right]\subset N,\left|\vec{m_{i}}\right|=\left|\vec{m_{j}}\right|=n$.

The result of each iteration of the Random Walk with Restarts is given by

(6) $$\begin{array}{c} p_{i}^{t\to \infty }=\overset{\infty }{\underset{t=0}{\prod }}\left(\left(1-r\right)W_{ij}p^{t}+rp^{0}\right), \end{array}$$

where in fact the algorithm stabilizes when the following condition is met:

(7) $$\begin{array}{c} \left(\vec{m_{1}}\left|{\vec{p}}^{t}-{\vec{p}}^{t-1}\right|\right)<\varrho , \end{array}$$

where $\vec{m_{1}}$ is disease mask vector and ${\vec{p}}^{t}$ is weight vector at time *t*. The product will result in a scalar that corresponds to the sum of the differences between two iterations. The condition is true when this value is less than a given constant *ϱ*.

### 2.5. Node Replacement

The recursive iteration of the cross subgraph mapping returns a new term. A node replacement strategy is used to replace the genes to be used. The selection of the node index *a*_*m*_ to be replaced by the node index *a*_*n*_ is given by the minimum value of *a*_*m*_ = min⁡_0≤*m*≤|*V*|_⁡*p*_*i*_^*t*→*∞*^ and where the candidate node is given by *a*_*n*_ = max⁡_0≤*i*≤|*V*|_⁡*W*_*ij*_∗*p*_*j*_^*t*→*∞*^.

## 3. Results and Discussion

In this section we explore and evaluate the performance of the proposed method. We present a systematic evaluation using a synthetic datasets based on well-known disease profiles. We also present how the results of RecRWR can be used to explore the resemblances mechanisms on breast cancer.

### 3.1. Validation Procedure

For each selected phenotype entry on the OMIM database we created a dataset with the associated genotypes. We have selected 100 phenotype diseases with at least 10 associated genotypes each. Then, we iteratively replace genes from the original dataset by random ones, in 20% increments, and the dataset is progressively converted to a fully random dataset. We use each of these protein datasets as seed nodes on the graph. We end up with a test space of 600 gene sets (6 random step levels plus 100 diseases).

### 3.2. Information Paradox

**Figure 1:**
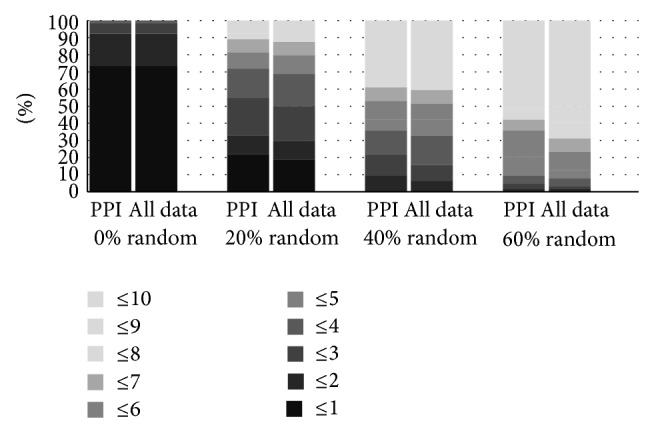
Comparison of the RWR method using PPI data and PPI enriched in biological terms.

Previous use of RWR on molecular biology typically concentrates on PPI networks. One would expect that including additional data would contribute to an improved overall result.  Figure 1 presents a comparison of the relative frequency of the ranks for each of the analysed datasets, for two of the tested methods (RWR over only PPI data and RWR over the whole network) and for four levels of randomness. From analysis of this graph it is clear there is no improvement with including external annotations on the original PPI network. Indeed for original dataset, with random effect, there are no perceptible differences between the two methods. This statement is even sharper when we test progressive levels of randomness. For instance, when 20% of the genes on the dataset are random, 55% of the RWR over PPI ranks the disease in the top 3, while with the RWR over all data this frequency drops to 48%. For 60% randomness, 35% of the RWR over PPI ranks the disease in the top 5, while with the RWR over all data the frequency drops to 23%. These results were the primary motivation for the work presented in this paper, as they clearly show that the RWR method is not suitable for dealing with multiple biological data.

### 3.3. RecRWR Results on Synthetic Datasets

**Figure 2:**
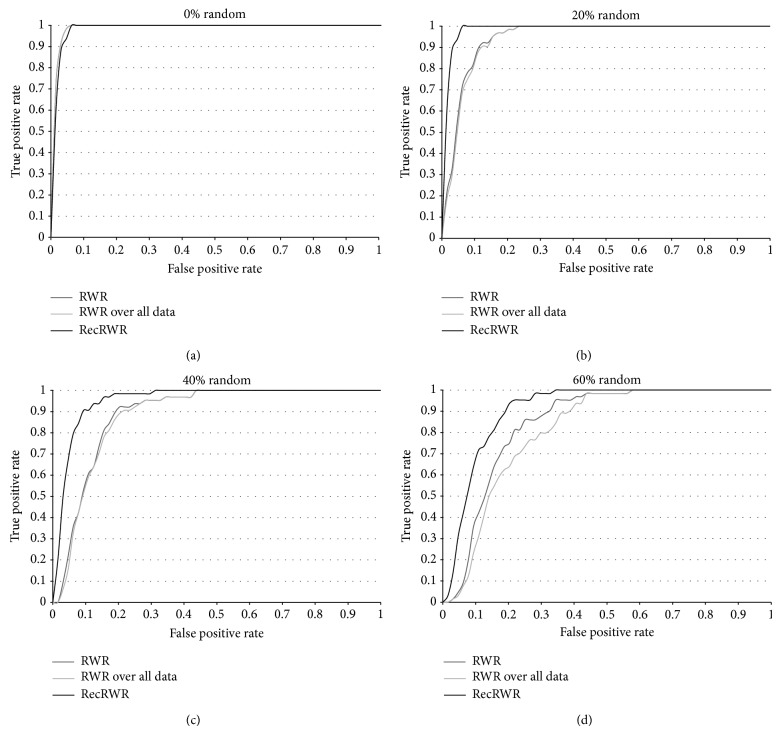
ROC curves with the comparison of the overall performance of RecRWR against existent methods.

We evaluate the performance of the RecRWR method using the receiver operating characteristic (ROC) curves where each curve contains the results for each level of randomness. A higher AUC (area under curve) corresponds to a better overall performance.  Figure 2 and Table 1 compile the obtained results.

With 0% randomness the AUC is approximately the same for the three methods, the proposed one having the lowest minimum value, which can be perceived visually. This means that in the absence of biological noise the protein annotation data does not contribute to improving the final result. However, if randomness is introduced the proposed method shows a strong improvement.

With 20% randomness the RecRWR AUC is 0.9834, which compared to 0.9453 on the RWR corresponds to a 4.0% of improvement. Comparing the behaviour of the RecRWR the 20% randomness reflects no real impact (−0.22%) on the obtained AUC.

For 40% and 60% the difference is even higher (7.1% for 40% and 7.6% for 60%) demonstrating the greater capability of the proposed method.

It is also relevant to note a 1.0 TPR (true positive rate, *y*-axis on the graphs from Figure 2), meaning that the disease is always correctly identified and is consistently obtained at the expense of a lower FPR (false positive rate, *x*-axis).

### 3.4. RecRWR Results on Breast Cancer

Breast cancer (MIM:114480) is considered a complex disorder having 23 known genotypes that are shared with other cancer-related disorders. We have used RecRWR over the common expression profiles of breast cancer to explore the network of diseases that share common mechanism. The diseases most closely related to breast cancer are hepatocellular carcinoma, bladder cancer, and lung cancer.

**Figure 3:**
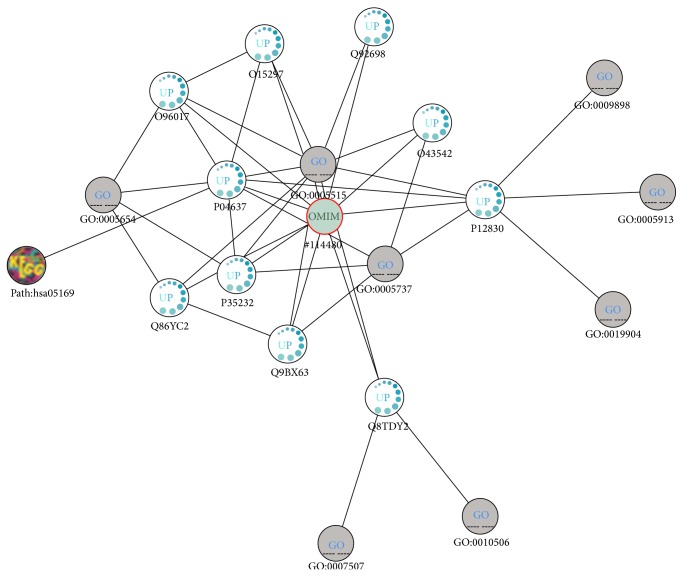
Network of biological concepts associated with breast cancer.

From analysing the network of associations, we can see that the proteins most related with breast cancer are responsible for important cellular functions, such as DNA repairing, cell cycle arrest and its regulation, induced cell death (apoptosis), and tumor suppression. Also, we can see that the more closely GO terms associated with breast cancer are protein binding and apoptotic process. This means that the probable causes of breast cancer are related to the impairment of all these functions. For instance, a genetic mutation causing loss of function on a tumor suppressor gene (such as the cellular tumor antigen p53, P04637) product would result in unrestrained cellular proliferation. Conversely, the transformation of a protooncogene (a gene that participates in a cell-growth pathway) into an oncogene (a protein that can induce cancer on animals) requires a gain-of-function mutation that will allow its permanent activation. An example of this is the epidermal growth factor receptor (EGFR, P00533), also present in Figure 3. EGFR is involved in the conversion of extracellular stimulus to cellular responses. Also, transcription errors are usually immediately corrected by DNA repairing proteins, like the DNA repair and recombination protein RAD54-like (Q92698), shown in the network. A mutation in this gene would result in the defective proteins, and subsequently the correction of transcription and translation errors would cease. Finally, the protein caspase-8 also seems to be a possible cause of breast cancer. Since caspase-8 is involved in the apoptotic process, impairment of this protein would result in the absence of apoptosis, and defective cells would not be destroyed.

The shortest path between the two diseases is mediated by the cellular tumor antigen p53. There are however other connections between the two nodes. For instance, the proteins receptor tyrosine-protein kinase erbB-2 (P04626), GTPase KRas (P01116), and caspase-8 (Q14790) also connect the two cancers. The influence of caspase-8 mutations on the onset of cancer was explained above. ERBB2 is a protooncogene, with the potential of being converted into an oncogene and inducing cancer. The GTPase KRas is involved in a great variety of important biological processes, including regulation of both of cell proliferation and gene expression, signal transduction, and cell signalling. The majority of the proteins analysed here are part of the same KEGG pathways: pathways in cancer (hsa05200), neurotrophin signaling pathway (hsa04722), and focal adhesion (hsa04510). The first pathway consists of an integration of the various cancer pathways. The neurotrophin signalling pathway is responsible for the differentiation and survival of neural cells. However, this second pathway is heavily regulated by other intracellular signalling cascades, in which some of the proteins presented in Figure 3 participate. The focal adhesion pathway plays important roles in the proliferation, differentiation, and survival of cells and in gene expression. In case of compromise of any of the proteins involved on this pathway cellular communication becomes defective, which can also result in cancer.

## 4. Conclusion

In this paper, we have proposed a graph-based approach to address the problem of selecting the best disease targets for multiconcept graphs. Towards this aim we build a multilayer biomedical graph that stores PPI data, annotated with stable knowledge from OMIM diseases and Biological Process from Gene Ontology. The inherent improvements of the proposed method are the use of multilayer networks formed with the PPI data and by the terms' associations; combination of this data to establish new associations among nodes; and use of degree-based methods for evaluating node weights.

Finally, we have presented comprehensive validation that demonstrates the superiority of the proposed approach, Recursive Random Walk with Restarts (RecRWR). The obtained results outline the superiority of the proposed approach in identifying disease candidates, especially with high levels of biological noise and benefiting from all data available.

## Figures and Tables

**Algorithm 1:**
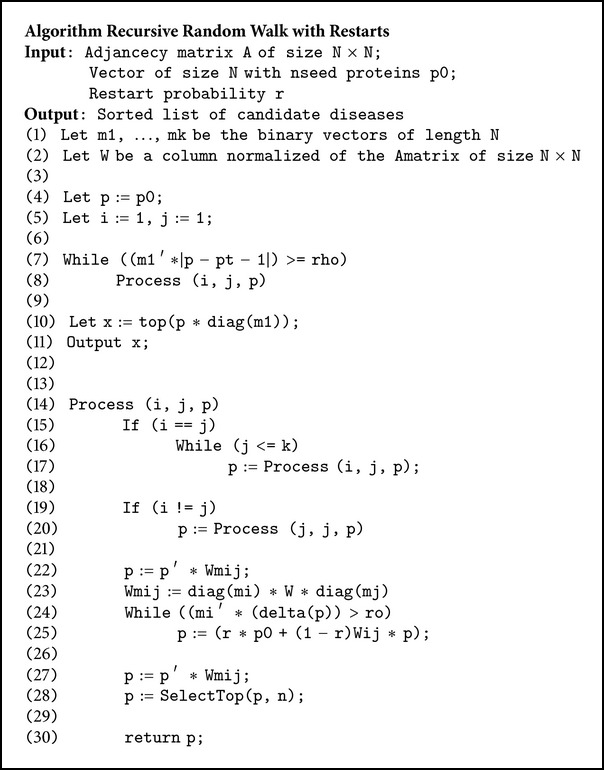
Pseudocode for the RecRWR method.

**Table 1:** Comparison of the AUC for the analysed methods.

	0%	20%	40%	60%
AUC-RWR	0.9866	0.9453	0.8894	0.8435
Δ (%)		−4.36%	−6.29%	−5.44%
AUC-RWR all data	0.9866	0.9417	0.8838	0.8115
Δ (%)		−4.77%	−6.55%	−8.91%
AUC-RecRWR	0.9856	0.9834	0.9534	0.9072
Δ (%)		−0.22%	−3.15%	−5.09%
